# Mental health symptoms are comparable in patients hospitalized with acute illness and patients hospitalized with injury

**DOI:** 10.1371/journal.pone.0286563

**Published:** 2023-09-20

**Authors:** Eve B. Carlson, Lisa Shieh, M. Rose Barlow, Patrick A. Palmieri, Felicia Yen, Thomas A. Mellman, Mallory Williams, Michelle Y. Williams, Mayuri Chandran, David A. Spain

**Affiliations:** 1 Dissemination and Training Division, National Center for Posttraumatic Stress Disorder, VA Palo Alto Health Care System, Department of Veterans Affairs, Menlo Park, California, United States of America; 2 Department of Psychiatry and Behavioral Sciences, Stanford University School of Medicine, Stanford, California, United States of America; 3 Department of Medicine, Division of Hospital Medicine, Stanford University School of Medicine, Stanford, California, United States of America; 4 Traumatic Stress Center, Summa Health, Akron, Ohio, United States of America; 5 Department of Surgery, Stanford University School of Medicine, Stanford, California, United States of America; 6 Georgetown Howard Universities Center for Clinical Translational Research, Washington, DC, United States of America; 7 Department of Psychiatry and Behavioral Sciences, Howard University College of Medicine, Washington, DC, United States of America; 8 Department of Surgery, Howard University College of Medicine, Washington, DC, United States of America; 9 Center of Excellence in Trauma and Violence Prevention, Howard University College of Medicine, Washington, DC, United States of America; VA Boston Healthcare System, UNITED STATES

## Abstract

**Background:**

High rates of mental health symptoms such as depression, anxiety, and posttraumatic stress disorder (PTSD) have been found in patients hospitalized with traumatic injuries, but little is known about these problems in patients hospitalized with acute illnesses. A similarly high prevalence of mental health problems in patients hospitalized with acute illness would have significant public health implications because acute illness and injury are both common, and mental health problems of depression, anxiety, and PTSD are highly debilitating.

**Methods and findings:**

In patients admitted after emergency care for Acute Illness (*N* = 656) or Injury (*N* = 661) to three hospitals across the United States, symptoms of depression, anxiety, and posttraumatic stress were compared acutely (Acute Stress Disorder) and two months post-admission (PTSD). Patients were ethnically/racially diverse and 54% female. No differences were found between the Acute Illness and Injury groups in levels of any symptoms acutely or two months post-admission. At two months post-admission, at least one symptom type was elevated for 37% of the Acute Illness group and 39% of the Injury group. Within racial/ethnic groups, PTSD symptoms were higher in Black patients with injuries than for Black patients with acute illness. A disproportionate number of Black patients had been assaulted.

**Conclusions:**

This study found comparable levels of mental health sequelae in patients hospitalized after emergency care for acute illness as in patients hospitalized after emergency care for injury. Findings of significantly higher symptoms and interpersonal violence injuries in Black patients with injury suggest that there may be important and actionable differences in mental health sequelae across ethnic/racial identities and/or mechanisms of injury or illness. Routine screening for mental health risk for all patients admitted after emergency care could foster preventive care and reduce ethnic/racial disparities in mental health responses to acute illness or injury.

## Introduction

Research has shown that 10–34% of patients presenting to emergency departments (EDs) with traumatic injury have posttraumatic stress disorder (PTSD) and/or depression when evaluated 3–12 months following the injury [1–6]. A substantial proportion of patients report symptoms of Acute Stress Disorder (ASD) [7, 8], a stress reaction occurring within two weeks of an event that is characterized by symptoms of PTSD and dissociation [9]. Anxiety disorders, while studied less than depression or PTSD, appear to occur at the same rate as depression (9%) and at a greater rate than PTSD (6%) [10]. Taken together, the existing literature on patients hospitalized with traumatic injury demonstrates that 30% or more may be at risk for persisting mental health problems [11].

Given that millions of patients are admitted with acute and sometimes life-threatening illness annually in the United States [12], it is important to know whether these patients are also at risk for mental health problems. The International Classification of Diseases (ICD-11) specifies life-threatening illness as a potential psychological traumatic stressor [13]. A theoretical framework for the impact of traumatic psychological events posits that the subjective experience of life threat can be traumatizing [14, 15]. Severe illness could also contribute to symptoms of depression and anxiety, whether or not the illness is a traumatic stressor.

While we know of no studies evaluating the mental health sequelae in patients admitted for care of acute illness, research on patients hospitalized in general medicine wards for emergency and non-emergency care in Australia found high rates of anxiety disorders (51%) and depression (44%) three months after discharge [16]. A study of U.K. Intensive Care Unit (ICU) patients found that 55% were above thresholds for anxiety (46%), depression (40%), or PTSD (22%) within one year of discharge [17]. Similarly, a study of U.S. ICU patients with respiratory failure or septic shock showed elevated depression in about one-third, and symptoms of PTSD in 7% [18]. Patients with narrowly defined conditions, such as myocardial infarction, have also been studied [19–21], but small samples and stringent inclusion/exclusion criteria limit generalizability of these studies to the larger population of patients admitted after emergency care in the U.S.

A high prevalence of mental health problems in patients hospitalized with acute illness would have significant public health implications. PTSD, depression, and anxiety can all be highly debilitating, impairing individuals’ quality of life through diminished occupational and role functioning [22–25], chronic medical problems [26, 27], disability [28, 29], increases in violence [30, 31], and suicidal behavior [32]. Globally, depression, PTSD, and anxiety contribute considerably to the burden of disease [33–36], and in a very large World Health Organization survey, life-threatening illness had the fourth highest lifetime prevalence of the potentially traumatic experiences studied [37]. In addition, in the United States and elsewhere, understanding patterns of mental health symptoms in specific racial/ethnic groups already at risk for poor health outcomes may inform symptom reduction or prevention strategies and provide opportunities to reduce racial/ethnic mental health disparities and improve mental health equity.

In the context of a study to develop a mental health risk screen for hospital patients, we examined whether acute and later mental health symptoms of ASD/PTSD, depression, and anxiety differed between patients treated in EDs and admitted who had acute illness (Acute Illness) and those who had injury (Injury). We studied acute symptoms and symptoms at two months post-discharge because studies on the trajectory of recovery in patients with traumatic injury have indicated that patients who have a low symptoms in the long term will have low symptoms acutely and at every subsequent time point [38]. We also examined whether the two groups differed in the number of mental health visits they received in the two months following hospital admission and whether symptoms differed in Acute Illness and Injury patients within groups who reported various racial/ethnic identities.

## Methods

### Participants

Patients were admitted through EDs at hospitals in Palo Alto, CA, Akron, OH, and Baltimore, MD, for acute illness or injury. All three hospitals are designated as Level I trauma centers. Level I trauma centers are tertiary care facilities that are verified as capable of providing care to the entire spectrum of injury patterns. Patients who self-identified as Asian, Native Hawaiian, or Pacific Islander were grouped as Asian, Asian American, and Pacific Islander (AAPI) for analysis; American Indian/Alaska Native patients were included in overall analyses but not in subgroup comparisons due to small sample size. Patients were classified as belonging to a group if that group was the sole ethnicity/race identified. Patients who endorsed more than one ethnicity or race were classified as Multirace.

### Measures

Data for this study were collected in the context of developing a risk screening measure. Therefore, all measures were self-report questionnaires that used uniform scales from 0 (“none of the time” or “not at all”) to 5 (“all or most of the time” or “4 or more times a day”). Based on data from the first 508 patients, acute measures of depression and anxiety were shortened in order to reduce the burden to hospitalized patients. Acute depression was assessed with 6 items from the Patient Health Questionnaire-8 (PHQ-8) [39] and acute anxiety was assessed with 2 items from the Generalized Anxiety Disorder-7 (GAD-7) [40] and three novel items. A detailed explanation of the development of these brief measures of depression and anxiety is provided in S1 File. The total scores for brief and full measures correlated very strongly (*r*s = 0.92–0.99) in the first 508 patients. Symptoms of ASD were assessed with 12 items from the Screen for Posttraumatic Stress Symptoms (SPTSS) [41] and 2 items from the Dissociative Symptoms Scale [42] to represent the 14 possible symptoms of ASD specified in DSM-5 [43].

At follow-up, symptoms of depression and anxiety were assessed with the PHQ-8 [44] and the GAD-7 [40]. Symptoms of PTSD were assessed with the 20-item SPTSS [41]. We defined elevation for depression and anxiety based on cut scores used in prior studies [40, 44] that were adjusted to reflect the response option scoring used in this study. Cut score values were 16 for the PHQ-8 and 15 for the GAD-7. Elevations for symptoms of PTSD at follow-up were calculated with a cut score of 16 based on mean and standard deviations in a subsample of adults from the community who reported lifetime exposure to no traumatic stressors [45].

### Procedure

Patients were enrolled between June 2018 and January 2021. All patients gave verbal informed consent after receiving a written explanation of the study and discussing it with a research assistant. The research assistant documented oral consent in study records. Waiver of documentation was approved by human subjects review panels at each site because the primary risk for the study was potential breach of confidentiality. This study was approved at the Stanford site by Stanford IRB 8, protocol 43236, approved 10/31/17; at the Akron site by Quorum, protocol 33238/3, approved 6/11/18; and at the University of Maryland Medical Center Baltimore site by Quorum, protocol 33238/2, approved 6/15/18.

In two hospitals, research assistants recruited English-speaking patients admitted after care in the ED. In the third hospital, bilingual, bicultural research assistants recruited patients who spoke English, Spanish, or Mandarin. Patients responded to questions asked in their preferred language on tablet computers, on paper forms, or orally, depending on their preferences and physical limitations. To maximize participation, multiple methods were used to contact patients two to three months post-admission. The methods included email and/or text message with a link to an electronic version of the questions, printed questionnaires sent by mail, or phone calls. Patients who provided email addresses were sent up to four emails. Patients who did not wish to receive emails were sent a questionnaire by mail. Patients who preferred to be contacted by text were also sent texts with links to the two-month questionnaire. If data were not received in response to emails, text, or a first mailed questionnaire, a second questionnaire was sent by mail. Patients were also called by phone if they did not respond to email, text, or mailed questionnaire.

## Results

Patients self-reported what type of acute illness or injury brought them to the hospital (Acute Illness = 656 patients [49.7%] and Injury = 661 [50.3%]). There were significantly more males (59%) admitted for injury and significantly more females (54%) admitted for acute illness (*Χ*^2^(2) = 22.92, *p* < .001). Patients’ ages ranged from 18 to 89 years old (mean = 49, *SD* = 17.4; median = 49; interquartile range = 29). Patients admitted after injury were older by about 3 years (*t*(1315) = -2.53, *p* = .01, *d* = -0.14). Half of the patients identified as a member of one or more ethnic/racial groups other than White (Table 1).

**Table 1:** Demographic information for patients with acute illness vs. injury.

Total	Acute Illness		Injury		Total	
	n	%	n	%	N	%
	656	49.7	661	50.3	1317	100
**Age in years (SD)**	47.6 (16.1)		50.1 (18.6)		48.9 (17.4)	
**Gender**						
Female	353	53.9	272	41.1	625	47.5
Male	301	46.0	390	58.9	691	52.5
Other	1	0.2	0	0.0	1	0.1
**Self-identified race**						
American Indian or Alaska Native	3	0.5	4	0.6	7	0.5
Asian & Pacific Islander	68	10.4	23	3.5	91	6.9
Black	181	27.6	129	19.5	310	23.5
Latinx	124	18.8	71	10.9	195	14.8
White	250	38.2	404	61.0	654	49.7
Multirace	29	4.4	29	4.4	58	4.4
Other	1	0.2	1	0.2	2	0.2
**Cause of hospital admission**						
Gastrointestinal or abdominal	238	36.3				
Sepsis/infection	83	12.7				
Cardiac	79	12.0				
Respiratory	74	11.3				
Neurological	40	6.1				
Unknown illness	18	2.7				
Other illness or pain ^a^	124	18.9				
Falls			250	37.8		
Vehicle crash			202	30.6		
Got attacked			65	9.8		
Hit by car while not in car			33	5.0		
Work injury			28	4.2		
Other injury ^b^			83	12.6		

^a^ “Other illness” includes blood sugar and diabetes complications, sickle cell disease, kidney problems, problems related to pregnancy, cancer, and other illnesses or conditions.

^b^ “Other injury” includes accidents during sports or hobbies, fires, animal bites, and other causes.

**Fig 1:**
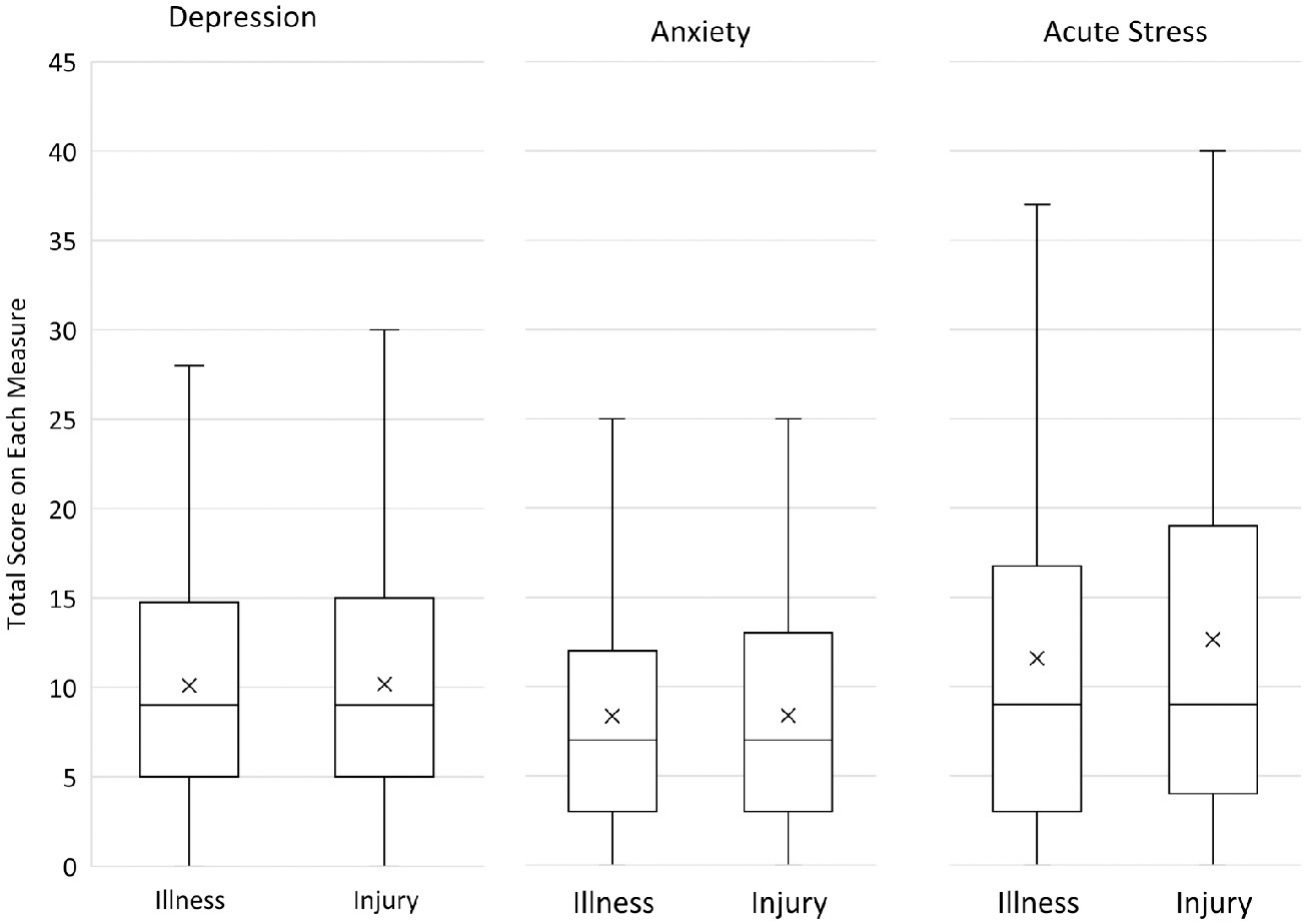
Medians, means, and interquartile ranges for acute mental health responses for patients admitted for acute illness (n = 656) and injury (n = 661). *Note*: *Means marked as X*.

**Fig 2:**
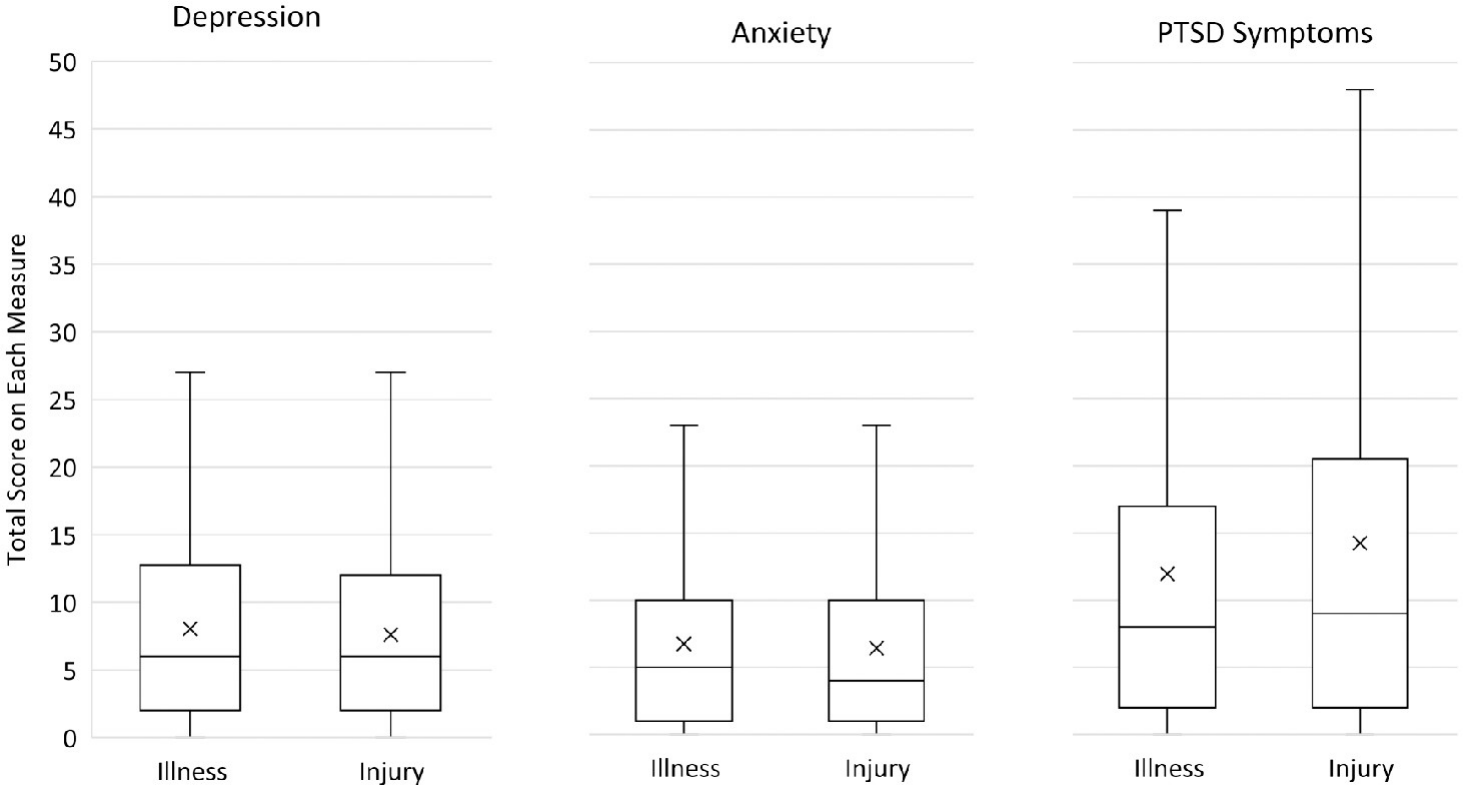
Medians, means, and interquartile ranges 2–3 months post-admission for patients admitted for acute illness (*n* = 416) and injury (*n* = 381). *Note*: *Means marked as X; PTSD = posttraumatic stress disorder*.

**Fig 3:**
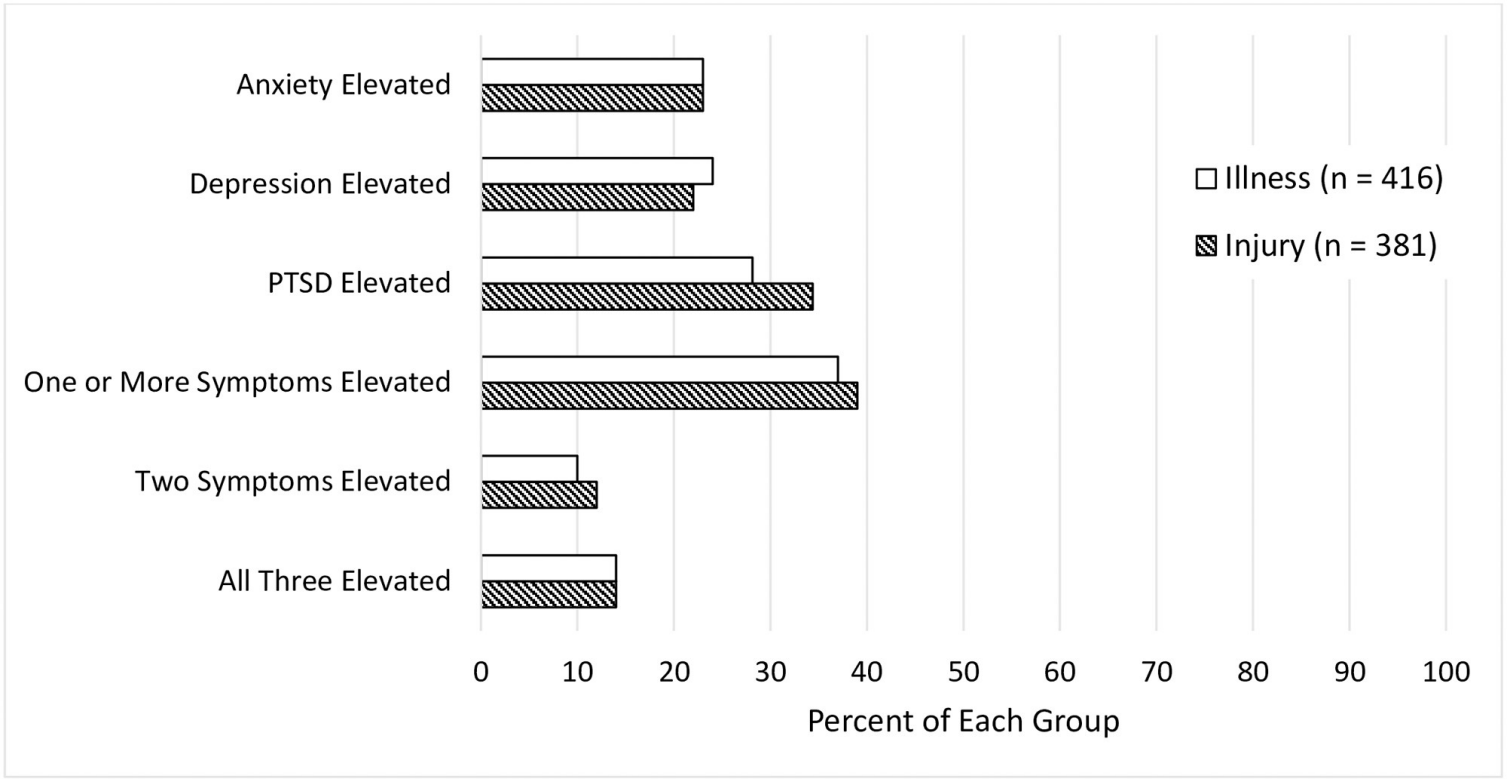
Similar proportions of acute illness (*n* = 416) and Injury (*n* = 381) groups had elevated symptoms 2–3 months post-admission. *Note*: *PTSD = posttraumatic stress disorder*.

Means and standard deviations for patients in the Acute Illness and Injury groups for acute symptoms of depression, anxiety, and ASD, and for depression, anxiety, and PTSD symptoms two months post-discharge are shown in Table 2 and Figs 1 and 2. As noted above, acute scores for each variable in Table 2 reflect responses to a subset of items used to assess the variables at two month post-admission. Two-sided t-tests showed no significant differences between Acute Illness and Injury groups on any acute or two-month post-admission symptom. Fig 3 shows the proportion of patients who reported elevated levels of depression, anxiety, PTSD symptoms, or combinations of these at two months after admission.

**Table 2:** Acute and two months post-admission mental health symptom means and standard deviations for patients with acute illness vs. injury by ethnic/racial group.

	Acute		2 Months Post-admission	
	Acute Illness	Injury	Acute Illness	Injury
**All patients**	**n = 656**	**n = 661**	**n = 416**	**n = 381**
Depression	10.1 (6.9)	10.2 (6.7)	8.1 (7.1)	7.6 (6.9)
Anxiety	8.4 (6.6)	8.4 (6.7)	6.7 (6.7)	6.4 (6.6)
ASD/PTSD symptoms	11.6 (11.3)	12.6 (11.6)	11.7 (13.6)	13.7 (15.7)
**AAPI**	**n = 68**	**n = 23**	**n = 44**	**n = 13**
Depression	10.1 (7.7)	9.4 (6.6)	7.5 (7.2)	4.4 (5.7)
Anxiety	5.7 (6.2)	8.2 (5.3)	5.9 (6.7)	2.5 (3.3)
ASD/PTSD symptoms	8.6 (9.1)	11.3 (9.5)	11.6 (13.9)	7.4 (10.8)
**Black**	**n = 181**	**n = 129**	**n = 101**	**n = 54**
Depression	9.3 (6.9)	10.2 (7.1)	7.7 (6.8)	8.6 (7.6)
Anxiety	8.4 (6.4)	9.2 (6.8)	6.9 (6.6)	7.3 (6.9)
ASD/PTSD symptoms	12.6 (12.0)	16.2 (12.5)*	11.6 (14.1)	20.7 (21.3)*
**Latinx**	**n = 124**	**n = 71**	**n = 67**	**n = 42**
Depression	10.2 (6.4)	10.6 (5.6)	7.4 (5.6)	7.9 (6.1)
Anxiety	9.0 (6.3)	9.8 (6.1)	5.6 (5.3)	8.0 (6.0)
ASD/PTSD symptoms	12.4 (10.4)	15.9 (11.7)	10.8 (10.3)	15.5 (13.6)
**Multirace**	**n = 29**	**n = 29**	**n = 15**	**n = 16**
Depression	11.5 (7.2)	10.8 (6.8)	8.8 (6.7)	9.4 (7.1)
Anxiety	9.7 (7.2)	9.7 (6.9)	5.2 (5.1)	8.2 (6.7)
ASD/PTSD symptoms	16.7 (16.7)	16.9 (14.3)	14.2 (15.0)	14.1 (14.0)
**White**	**n = 250**	**n = 404**	**n = 187**	**n = 256**
Depression	10.4 (6.8)	10.0 (6.8)	8.4 (7.7)	7.4 (7.0)
Anxiety	8.5 (6.7)	7.7 (6.7)	7.3 (7.1)	6.1 (6.6)
ASD/PTSD symptoms	10.5 (10.6)	10.5 (10.6)	11.7 (14.0)	12.2 (14.6)

* = t-test is significant using Bonferroni correction (alpha of .01)

Note: American Indian/Alaska Native patients are included in “all patients” but not in a separate group for analysis, as they were less than 1% of the sample. Numbers in columns are mean values. Numbers in parentheses are standard deviations.

Means and standard deviations for acute and two-month post-admission symptoms by ethnic/racial identity are shown in Table 2. Two-sided t-tests compared mean symptoms for the Acute Illness and Injury groups within each ethnic/racial identity category with alpha = .01 to correct for multiple comparisons. The only significant differences were that Black patients in the Injury group had higher ASD (*t*(308) = -2.52, *p* = .006, *d* = -0.29) and higher 2-month PTSD symptoms (*t*(153) = -3.19, *p* < .001, *d* = -0.54) compared to Black patients in the Acute Illness group.

In further analyses, we found that 29% of Black in the Injury group reported having been physically assaulted, compared to 5% of Injury patients with other ethnic/racial identities. In Black patients who were assaulted, 76% percent of injuries were due to gunshots or knife wounds and 24% were the result of being hit or beaten with fists or a weapon. Of all patients assaulted, 58% were Black, whereas Black patients made up only 19% of the Injury group. Scores on the PTSD measure at two months for the 65 people who were assaulted were double those of patients who had other mechanisms of injury (*M* = 26.9, *SD* = 16.31 vs. *M* = 12.8, *SD* = 15.32). For this comparison, *p* < .001 (*d* = 0.91).

There was no difference (*Χ*^2^(1) = 1.01, *p* > .05) in the proportion of each group that received mental health care during the 2–3 months post-admission, with 11% of Acute Illness and 13% of Injury group patients who completed the follow-up reporting that they received at least one session of mental health care. Of patients who had at least one type of symptom elevation at follow-up (*n* = 305), there was also no difference between Acute Illness (22%) and Injury (23%) groups in the proportion of patients who had received mental health care in the previous 2–3 months (*Χ*^2^(1) = 0.006, *p* > .05).

The overall follow-up rate was somewhat lower for Injury group patients (58%) than for Acute Illness group patients (63%) (*Χ*^2^(1) = 4.60, *p* < .05). There was differential retention based on ethnic/racial group, with follow-up rates ranging from a high of 68% in White patients to a low of 50% in Black patients (*Χ*^2^(5) = 38.10, *p* < .001). There was also differential retention based on acute symptom severity. Of patients who scored in the top half of all acute symptoms, 53% completed follow-up, compared to 69% of patients who scored lower on acute symptoms (*Χ*^2^(1) = 33.44, *p* < .001). Patients who completed the follow-up also had less financial stress (*M* = 3.17, *SD* = 2.22) than did patients who did not complete (*M* = 3.98, *SD* = 2.29), *t*(1222) = 6.18, *p* < .001, *d* = 0.36.

## Discussion

Research on mental health status of patients hospitalized after emergency care has focused largely on patients with traumatic injury [1, 4, 46]. This study is the first to our knowledge to report that U.S. patients hospitalized after emergency care for acute illness have comparable levels of mental health symptoms. Acutely and two months after admission, mental health symptoms of patients with Acute Illness were high and did not differ from those of patients with Injury. Elevated levels of depression, anxiety, and/or PTSD symptoms were reported two months after admission by 39% of patients with Injury and 37% of patients with Acute Illness. Two months after admission, comparable proportions of patients with Acute Illness and patients with Injury were found to have elevated levels of anxiety (23% for both groups), depression (24% and 22%, respectively), and PTSD symptoms (28% and 34%). The overall pattern of responses was similar in both groups: just over half of the patients either did not endorse symptoms or recovered from their symptoms within 2 months, while the other participants in both groups had notably high levels of mental health symptoms at both time points.

Compared to a prior study of mental health at two months post-injury in traumatic injury patients, we found a lower proportion of injured patients with elevated depression symptoms and a comparable proportion with elevated PTSD symptoms [47]. We found lower proportions of patients with elevated anxiety and depression for patients with acute illness than did a study of Australian patients hospitalized in general medicine wards that reported 51% had elevated anxiety and 41% had elevated depression three months post-discharge [16]. Difference in the rates across studies may be due to differences in the populations studied and/or to differences in measures used and how elevations were defined. Similar to earlier research [18], we found that mental health symptom types tended to co-occur. Only 13–14% of participants were elevated on just one mental health symptom type at 2 months, compared to 23–26% having two or more symptom types (see Fig 3). For this reason, it is important to keep in mind that detecting one dimension of distress may be just the tip of the iceberg.

Comparison of mental health symptoms reported by patients with acute illness and patients with injury within ethnic/racial groups showed no differences for most symptoms in most groups. The only differences observed were significantly higher levels of ASD and PTSD symptoms reported by Black patients with injury compared to Black patients with acute illness. Further analyses indicated that a much higher proportion of Black patients had experienced assaults (29%) compared to patients with other ethnic/racial identities (5%) and PTSD scores were twice as high for patients who had been assaulted compared to patients with injuries from other causes. The higher assault rates among Black patients may have contributed to higher ASD and PTSD rates observed in this study as assaults have been found to be associated with higher conditional risk of PTSD than injury from other causes [48]. Assaults may be more traumatizing than other injuries because they typically involve a higher threat of injury or death and challenge assumptions about safety and the trustworthiness of others [49, 50]. Higher assault rates may reflect patient environments with higher rates of violent crime. Analyses of the same dataset found that social determinants of health, such as financial stress and discrimination, were worse in Black patients and were associated with mental health disparities [51]. Given that interpersonal violence is associated with both physical wounds and mental health problems, these patients require medical interventions targeted at both physical and mental health symptoms.

Strengths of this study include its enrollment and study site diversity, longitudinal design, systematic identification and recruitment of all eligible patients, inclusion of patients whose preferred language was English, Spanish, or Chinese, broad inclusion criteria, and success enrolling patients with a wide range of ethnic and racial identities. The broad inclusion criteria make it possible for our findings to be generalized to a wider population of hospital patients admitted after emergency department care at hospitals with Level I trauma centers. An additional strength was the large samples of patients with acute illness or injury, which provided excellent power to detect any clinically relevant differences between groups.

There were also limitations to the study. Like all studies with voluntary participants, there may have been selection bias if patients who participated in the study differed on the variables studied from patients who declined to participate. Within the groups of patients studied, patients were heterogenous in terms of the illness or injury they were treated for, and the samples may not have reflected the population of patients hospitalized in the United States. Use of alternative response options for measures of anxiety and depression is a limitation because it means scores on the measures cannot be compared to scores on the measures in other studies. It was also a limitation that the definition of elevations used was not based on data from non-clinical samples.

Despite considerable efforts to reach patients, the overall follow-up rate of 61% at two months was lower than desirable. In a prior study at one site, the follow-up rate for injury patients was 76% [6], which is considerably higher than the rate of 57% for the same population of patients at that site in this study. We experienced several obstacles to collecting follow-up data in this study, including patients’ reluctance to answer cell phones due to frequent spam calls, patients’ phone numbers no longer in service, and patients moving between enrollment and follow-up. Our findings of higher levels of acute symptoms and financial stress in patients lost to follow-up indicate that these may also have been impediments to retention. Therefore, sampling bias may underestimate the actual levels of symptoms. Future studies may improve follow-up rates by increasing efforts to address these obstacles.

Our findings have implications for public health and the care of patients admitted after emergency care for acute illness or injury. Surgeons, nurses, social workers, and others who treat patients in trauma centers are generally aware of the mental health risks of traumatic injury. The American College of Surgeons’ Resources for the Optimal Care of the Injured Patient sets the standards by which U.S. trauma centers are verified and now specifies that all Level I and Level II trauma centers must meet the mental health needs of their patients by having a structured approach to identify patients at high risk of mental health problems and a process for referral to a mental health provider [52]. A recent survey of U.S. trauma centers indicates that 28% of trauma centers reported that they routinely screen patients for PTSD and 38% reported that they routinely screen for depression [53]. General inpatient medicine teams, including hospitalists, social workers, case managers, and nurses, also need to be aware of mental health risk for patients they treat.

Our results suggest that medical patients with acute illness requiring admission through the ED could also benefit from routine screening for mental health risk. The US Preventive Services Task Force guidelines recommend screening for depression [54], but this is not currently required in hospital settings. In a report on quality of Medicaid care in 2020, only 20% of U.S. states reported depression screening as a quality measure [55]. Given the low rates of mental health care during the 2–3 months post-admission in patients with acute illness we studied, it appears that the mental health needs of these patients are not being met. Routine mental health risk screening conducted during hospitalization as part of discharge planning could identify those at risk and connect them to mental health resources. Such screening could also foster research on posttraumatic mental health problems and efforts to reduce or prevent them. We have reported elsewhere on our work to develop and cross-validate a screen for mental health risk for all hospital patients admitted after emergency care [56]. The Hospital Mental Health Risk Screen provides accurate assessments of mental health risk for acute illness and injury patients from diverse ethnic/racial backgrounds [56].

Future research should investigate whether acutely ill patients who are hospitalized in other countries show similar elevations in depression, anxiety, and PTSD. Studies are also needed to determine whether mental health symptoms in patients with acute illness and injury are associated with longer lengths of stay, increased hospital readmission rates, and/or increased health care costs [57]. Similarly, untreated mental health problems may contribute to readmissions and increased healthcare costs. Understanding patterns of mental health symptoms in specific racial and ethnic groups already at risk for high hospital readmission may inform reduction or prevention strategies. Lastly, interventions found to be effective for injured patients should be studied for their effectiveness in patients with acute illness.

## Conclusion

Two months post-admission, almost half of patients with Acute Illness and patients with Injury had elevated mental health symptoms. The study findings of high and similar levels of mental health symptoms in patients with Acute Illness compared to patients with Injury indicate there has been under-recognition of the mental health needs of patients with acute illness. The large number of such patients in the U.S. and globally suggests that the burden of unmet mental health needs is great. The finding of significantly higher symptoms and interpersonal violence injuries in Black patients with injury suggests that there may be important and actionable differences in mental health sequelae across patients with different ethnic/racial identities and/or different mechanisms of injury or illness. Findings of high and similar levels of mental health symptoms in patients with acute illness or injury and higher symptoms in patients with interpersonal violence injuries could both be addressed by routine mental health risk screening of patients admitted after emergency care.

## Supporting information

S1 File Summary of analyses to reduce items assessing acute depression and anxiety. (DOCX)
