# Supplementary material for: Mental health symptoms are comparable in patients hospitalized with acute illness and patients hospitalized with injury
Source: PLoS One. 2023 Sep 20;18(9):e0286563. doi: 10.1371/journal.pone.0286563 (PMC10511104; doi:10.1371/journal.pone.0286563)
Supplement: S1 File — (DOCX) [file pone.0286563.s001.docx]

**Supplemental File 1: Summary of Analyses to Reduce Items Assessing Acute Depression and Anxiety**

Analyses were conducted to select smaller sets of depression and anxiety items while retaining precision in measurement for these constructs. For the anxiety construct, we also examined data from novel items that used everyday language to describe anxiety. In addition, we sought to use empirical methods to select items to assess depression and anxiety as affects, rather than lists of symptoms based on diagnostic criteria. Both the PHQ-9 and GAD-7 items were not empirically derived, but were based on the DSM-IV diagnostic criteria for depressive disorders (Kroenke 2001) and for Generalized Anxiety Disorder (Spitzer 2006).

Item data were analyzed for N = 508 patients. For anxiety, items were the seven GAD-7 items and three novel items: “Feeling very anxious or worried”, “Feeling very stressed”, and “Feeling overwhelmed.” For depression, items were the first 8 items of the PHQ-9. We conducted factor analyses (FAs) and forward regressions on depression items and anxiety items to identify those most strongly associated with the underlying constructs. Candidate items for elimination were items that did not load highly in FAs, items that did not contribute much to prediction (in regression analyses), and items that were endorsed at unusually low rates (20% or fewer participants endorsed more than “a little”) or unusually high rates (40% or more endorsed “all or most of the time”).

Once candidate items for elimination were identified, total scores for retained items were correlated with total scores on original construct measure in each racial/ethnic group. Final determinations about how many items to retain were based on these correlations. In each measure, enough items were retained to maintain a correlation of .92 or higher with the full set of items.

**Correlations between Brief and Full Measures of Risk Factors Across Race/Ethnicity**

|  | Asian  (n=23) | Black  (n=113) | Latinx  (n=41) | Multiracial or Other  (n=20) | White  (n=311) |
| --- | --- | --- | --- | --- | --- |
| 6-item & 8-item depression | .99 | .98 | .98 | .99 | .98 |
| 5-item & 10-item anxiety | .95 | .93 | .96 | .92 | .93 |

**Items retained:**

**Depression:**

Feeling down, depressed, or hopeless (PHQ-8 item 2)

Trouble falling or staying asleep, or sleeping too much (PHQ-8 item 3)

Feeling tired or having little energy (PHQ-8 item 4)

Poor appetite or overeating (PHQ-8 item 5)

Feeling bad about yourself -- or that you are a failure or have let yourself or your family down (PHQ-8 item 6)

Trouble concentrating on things, such as reading or watching TV (PHQ-8 item 7)

**Anxiety:**

Feeling very anxious or worried (novel item)

Feeling very stressed (novel item)

Feeling overwhelmed (novel item) Not being able to stop or control worrying (GAD-7 item 2)

Becoming easily annoyed or irritable (GAD-7 item 6)

**Items not retained:**

**Depression:**

Little interest or pleasure in doing things (PHQ-8 item 1)

Moving or speaking so slowly that other people could have noticed? Or the opposite - being so fidgety or restless that you have been moving around a lot more than usual (PHQ-8 item 8)

**Anxiety:**

Feeling nervous, anxious, or on edge (GAD-7 item 1)

Worrying too much about different things (GAD-7 item 3)Trouble relaxing (GAD-7 item 4)

Being so restless that it's hard to sit still (GAD-7 item 5)

Feeling afraid as if something awful might happen (GAD-7 item 7)
